# The BRG1 ATPase of human SWI/SNF chromatin remodeling enzymes as a driver of cancer

**DOI:** 10.2217/epi-2017-0034

**Published:** 2017-05-19

**Authors:** Qiong Wu, Jane B Lian, Janet L Stein, Gary S Stein, Jeffrey A Nickerson, Anthony N Imbalzano

**Affiliations:** 1 Department of Pediatrics, University of Massachusetts Medical School, 55 Lake Avenue North, Worcester, MA 01655, USA; 2 Department of Biochemistry, University of Vermont College of Medicine, 89 Beaumont Avenue, Burlington, VT 05405, USA; 3 Department of Biochemistry & Molecular Pharmacology, University of Massachusetts Medical School, 364 Plantation Street, Worcester, MA 01605, USA

**Keywords:** ADAADi, breast cancer, BRG1, BRM, cancer metabolism, chromatin remodeling, drug transporters, mammalian SWI/SNF enzymes, PFI-3

## Abstract

Mammalian SWI/SNF enzymes are ATP-dependent remodelers of chromatin structure. These multisubunit enzymes are heterogeneous in composition; there are two catalytic ATPase subunits, BRM and BRG1, that are mutually exclusive, and additional subunits are incorporated in a combinatorial manner. Recent findings indicate that approximately 20% of human cancers contain mutations in SWI/SNF enzyme subunits, leading to the conclusion that the enzyme subunits are critical tumor suppressors. However, overexpression of specific subunits without apparent mutation is emerging as an alternative mechanism by which cellular transformation may occur. Here we highlight recent evidence linking elevated expression of the BRG1 ATPase to tissue-specific cancers and work suggesting that inhibiting BRG1 may be an effective therapeutic strategy.

## Mammalian SWI/SNF complexes are chromatin remodeling enzymes

*In vitro* biochemical approaches demonstrated that mammalian SWI/SNF complexes altered the structure of reconstituted chromatin particles in an ATP-dependent manner and made chromatin more accessible for transcription factor binding [1–3]. SWI/SNF enzymes associate with chromatin via protein:protein and nonspecific protein:chromatin interactions [4]. Work on the enzymatic mechanism of ATP-dependent chromatin remodeling has been an ongoing endeavor and has been summarized elsewhere [5–8]. Evidence that mammalian SWI/SNF enzymes altered cellular chromatin was demonstrated by changes in nuclease accessibility upon experimental manipulations to block the association of the enzyme with the transcriptional machinery or to express an enzymatically dead ATPase [9,10]. Over the last 15 or so years, biological roles for mammalian SWI/SNF enzymes and the individual subunits have been established in development and tissue differentiation as well as in response to signaling mechanisms of many kinds [11–14]. Mammalian SWI/SNF-mediated chromatin remodeling has effects on transcription, replication, repair and recombination, though research in the area of regulation of gene expression has been the most extensively pursued. While it is known that mammalian SWI/SNF enzymes regulate some constitutively expressed genes [15], enzyme activity is most closely linked to changes, or in some cases, reprogramming of gene expression in response to developmental, environmental or other signaling cues.

One of the more remarkable properties of the mammalian SWI/SNF enzymes is the heterogeneity of enzyme composition. Several of the subunits derive from different genes that encode similar but distinct proteins, splice variants for some subunits exist and different subunits show preferential association or mutual exclusivity with others [16]. Few of the subunits are found as independent proteins or as part of other protein complexes. Of particular relevance is the finding that there are two highly related but mutually exclusive ATPases, called BRM and BRG1, that act as the catalytic subunit for mammalian SWI/SNF enzymes [1–3,17,18]. The ATPases belong to the SNF2 family of DNA-dependent ATPases that are related to DExx-box helicases, yet these proteins show no helicase activity [19,20]. *In vitro*, BRG1 and BRM have similar biochemical properties [21,22], but in cells they have both overlapping and differing roles [23–26]. The functional specificities, and even the biological needs for differently assembled enzyme complexes, remain an enigma.

**Figure 1.**
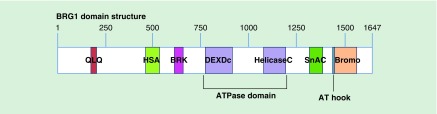
**Domain structure of BRG1.** Conserved domains are labeled. Numbers across the top of the schematic represent amino acids.

The catalytic ATPase subunits, BRG1 and BRM, are multidomain proteins that contain both DNA and protein interaction modules (Figure 1). These homologs share 86% similarity at the protein level [27]. Among the conserved protein domains is the ATPase domain that defines the broader family of SNF2 ATPases, which translate the energy generated by ATP hydrolysis to mechanical motion on DNA templates [20]. Fine structure analysis of the ATPase domain by both mutational structure-function analyses and crystallography has provided details about ATP binding and hydrolysis that reflect the common mechanism of action of this domain among the SNF2 ATPase family members [19]. The function of the BRK domain is unknown, but the presence of this domain is associated with helicases and transcription factors [28]. QLQ domain function is also unknown but has been postulated to mediate protein:protein interactions [29,30]. The HSA domain mediates intracomplex protein:protein interactions between BRG1 and the BAF250a/ARID1A subunit and is required for BRG1-dependent transcriptional activation by nuclear hormone receptors [31]. This domain is also required for interactions with the Ku70 protein, which links BRG1 with topoisomerase 2β and PARP1 as part of the activation complex necessary for nuclear hormone-mediated gene activation [32]. Three domains contribute to chromatin binding. The Snf2 ATP coupling (SnAC) domain is conserved among Snf2 ATPases but has been characterized only in the yeast Snf2/Swi2 protein. SnAC and ATPase domains directly bind to the histone proteins when SWI/SNF enzymes are bound to nucleosomes, and the SnAC domain is essential for ATPase and chromatin remodeling activities [33,34]. The AT hook is a nonspecific DNA binding domain [35]. Bromodomains [36] are 110 amino acid domains that are found in many chromatin-associated proteins. Bromodomains can interact specifically with acetylated lysines present on histone H3 and H4 tails [37,38].

## Mammalian SWI/SNF enzymes are linked to cancer

The first definitive link between mammalian SWI/SNF enzymes and cancer came from a seminal study identifying the loss of the subunit called INI1/hSNF5/BAF47 as causal for development of pediatric rhabdoid tumors [39]. Early mouse studies indicated that Ini1 as well as the Brg1 ATPase subunit were tumor suppressor proteins [23,40–43]. These findings were consistent with cell culture-based and subsequent mouse modeling studies finding functional interactions between BRG1 and numerous cell cycle regulatory proteins, including Rb, p53 and others [13,44,45]. Collectively, the data indicated that mammalian SWI/SNF enzymes normally contribute to cell cycle regulation and that loss of specific subunits and/or function result in cell cycle defects that could lead to tumor formation.

More recent studies using global approaches to identify mutations associated with cancer have revealed that human SWI/SNF enzyme subunits and proteins that associate with these subunits are mutated in approximately 20% of all human cancers [46,47], with suggestions that the actual frequency may be higher [48]. Loss or mutation of BRG1 has been documented in a number of cancers, including, but not limited to, lung, small cell carcinoma of the ovary, hypercalcemic type, medulloblastoma and Burkitt's lymphoma. These findings have led to discussions of strategies to reverse the effects of mutations, especially mutations that result in silencing of the expression of one or more subunits, as a novel epigenetics-based approach to cancer therapy [48–50]. In addition, considerable attention has been given to the idea of inducing synthetic lethality; targeting the BRM ATPase in cancers already containing nonfunctional BRG1 may be an effective strategy to treat such tumors [51–53]. Note, however, that some tumors, including lung and small cell carcinoma of the ovary, hypercalcemic type, lack both BRG1 and BRM [54–56], providing additional complexity to rational design approaches to restoring BRG1 or BRM function.

While the idea that mutation or loss of SWI/SNF subunit proteins, because they are ubiquitously expressed and are essential contributors to gene expression, replication, repair and recombination, will lead to cellular transformation is well established, it is important to remember that mammalian SWI/SNF enzyme function is highly context-dependent. The enzymatic activity generates changes in chromatin accessibility, which can either negatively or positively affect chromatin utilization. Thus, it should not seem unexpected that overexpression of SWI/SNF subunits may similarly cause initiation or acceleration of cancer progression. In this review, we will concentrate on an emerging theme: overexpression of the BRG1 ATPase is correlated with tumorigenesis.

## BRG1 is unlikely to be a tumor suppressor for breast cancer

The initial evidence suggesting that BRG1 was a tumor suppressor protein came from knockout mouse studies. A null mouse was embryonic lethal, while heterozygotes presented with mammary carcinomas [23,40]. At issue was the relatively low frequency (9%) of mammary tumor incidence, and subsequent work indicating that mammary-specific genetic depletion of the gene encoding BRG1 did not result in mammary tumors [40,57]. Initial exome sequencing of 507 primary breast tumors failed to identify any tumors with mutant BRG1 [58], while later analysis of the TCGA database indicates less than 2% mutation frequency [48].

These potentially puzzling observations were supported by other work using human cell lines. Knockdown of BRG1 in mammary epithelial cells slowed the rate of proliferation [59], instead of inducing more aggressive characteristics of a cell type that had lost a tumor suppressor. Knockdown of BRG1 in breast cancer cells similarly resulted in a reduction in the rate of proliferation in culture [60,61] and in orthotopic xenografts [61]. Complete knockout of BRG1 by CRISPR/CAS9 technology resulted in cell death, affirming a requirement for BRG1 in breast cancer cell viability [61]. Immunohistochemistry studies of primary breast tumors showed elevated BRG1 expression in 35% to nearly 100% of samples [60–62], cementing the conclusion that elevated BRG1 expression correlates with breast tumorigenesis. Importantly, high BRG1 expression was correlated with poor overall patient survival [60–62], raising the possibility that BRG1 could be used as a prognostic indicator. In particular, Do et al. have argued that high BRG1 expression levels in invasive ductal carcinoma patients are a predictive marker for patients at high risk of developing metastases [62].

## BRG1 likely promotes breast cancer cell proliferation through multiple mechanisms

Triple negative breast cancer is characterized by the absence of the estrogen receptor (ER), the progesterone receptor (PR) and low to normal levels of HER2, a receptor tyrosine kinase encoded by the *ERBB2* gene that is often amplified or overexpressed in breast cancer. Absence of these markers means that the use of therapeutic approaches targeting these markers is not possible, and patients can only be treated by less specifically targeted cytotoxic drugs. Our recent work has demonstrated that BRG1 promotes triple negative breast cancer cell proliferation via multiple mechanisms. First, BRG1 promotes lipid, and specifically, fatty acid synthesis in support of cell proliferation [63]. Tumor cells typically use *de novo* fatty acid synthesis pathways even when exogenous fatty acids are available [64], and key enzymes in fatty acid and lipid synthesis are frequently overexpressed in breast cancer [65,66]. Knockdown of BRG1 in triple negative breast cancer cells substantially lowered *de novo* lipid synthesis, which correlated with decreased cell proliferation. Subsequent investigation determined that BRG1 upregulates expression of enzymes responsible for fatty acid and lipid biosynthesis and likely does so in a direct manner, as BRG1 binds at the loci encoding these genes [63]. The generality of these findings in other types of breast cancer awaits further study.

Our work also demonstrated that BRG1 upregulates ATP-binding cassette (ABC) transporter expression in response to drug treatment [67]. ABC transporters comprise a large family of highly conserved, ATP-dependent, membrane-associated protein complexes that perform cellular import and export of numerous substrate molecules. Some ABC transporters are of great importance in cancer treatment because chemotherapeutic drugs can induce transporter expression, which can lead to increased export of and resistance to the drug [68]. Recent reports showed that BRG1 knockdown increased chemosensitivity to an assortment of chemotherapeutic drugs currently used in the clinic [67,69–72]. Some of these results were attributed to BRG1-dependent induction of ABC transporter expression [67,71]. Knockdown of BRG1 abrogated transporter induction, and, importantly, increased the intracellular concentration of the drugs, which explains the observed chemosensitivity [67].

ER and/or PR positive breast cancers may be regulated by BRG1 by additional mechanisms. BRG1 associates with ER and is required for ER-mediated transcriptional activation [73–75]. Similarly, progestin-induced gene activation involves BRG1 and the SWI/SNF enzyme complex [76,77]. It is possible that BRG1 contributes to cancer progression driven by dysregulated ER and/or PR signaling, but exact mechanisms remain to be determined.

## BRG1 has a positive role in promoting proliferation in other cancer cell types

BRG1 is required for proliferation of HeLa cells via its regulation of p53 function [78]. Other works concluded that acute myeloid leukemia cells require BRG1 for proliferation and survival [79,80]. BRG1-dependent survival was linked to BRG1-dependent chromatin remodeling function at the *MYC* locus that resulted in hematopoietic transcription factors binding to enhancer sequences and the formation of a loop between the factor-bound enhancer and the promoter, which stimulated *MYC* gene expression [79]. Small cell lung cancer tumor cells that contain mutations in the Myc-associated factor MAX also require BRG1 for proliferation and survival [81]. In contrast, BRG1 knockdown in small cell lung cancer cells with wildtype MAX showed no effect on proliferation or viability, leading to the conclusion that BRG1 function is necessary for cell survival in the absence of functional MAX. These data, like those described for breast cancer, are not consistent with BRG1 functioning as a classical tumor suppressor.

## BRG1 is expressed at elevated levels in other tumor types

### Melanoma

Melanocytes, pigment-producing cells, exist in a number of locations in the body but are best known for protecting the epidermis against the harmful effects of UV radiation. Melanocytic neoplasms originate from neural crest-derived melanocytes and can range from benign melanocytic naevi to malignant melanoma, which is considered to be the most aggressive form of skin cancer. The development and progression of melanoma have been attributed to independent or combined genetic and epigenetic events involving the RAS/RAF/MAPK, JNK, PI3K/Akt and Jak/STAT signaling pathways. Microphthalmia-associated transcription factor (MITF), the lineage determinant that drives expression of melanocyte-specific genes, is also implicated in melanocyte transformation [82,83].

There appears to be some redundancy in the function of BRG1 and the related BRM ATPase in promoting melanoma cell proliferation; knockdown of both, or of one if only one is present, blocks cell division [84–86]. Investigation of BRG1 and BRM expression in primary tumors resulted in clear evidence of high levels of both in primary as well as in metastatic melanomas [84,87]. Mutation of BRG1 appears to be rare [88]. Patient survival data indicate a trend showing that low to moderate BRG1 expression improves short-term survival, but to date there is no statistical correlation between BRG1 expression and long-term patient survival [84]. Based on correlation between BRG1 expression and cell cycle regulators affected by BRG1, it has been proposed that the critical BRG1 function is in the initiation stages of melanoma progression [84], though it appears that BRG1, and possibly BRM, function contributes throughout melanoma progression [87].

The mechanism of SWI/SNF function in melanoma appears to be predominantly through regulation of gene expression. MITF requires functional SWI/SNF ATPases to promote melanocyte differentiation [89]. Similarly, the SWI/SNF ATPases promote MITF-driven transcription in melanoma cells and further add to the increased transcriptional activity of MITF target genes by stimulating increased expression of MITF itself [85,86]. Genes altered by manipulation of BRG1 and BRM expression include those affecting cell proliferation and survival, consistent with observed changes in cell cycle progression [84–86]. BRG1 and MITF physically interact and associate at melanocyte-specific promoters [86,87,90] as well as at the MITF promoter [85], suggesting direct contribution to target gene expression. Protection against UV-induced cell death specifically requires BRG1 to activate an inhibitor of apoptosis that is a transcriptional target of MITF [91]. SWI/SNF ATPases also promote the expression of prosurvival genes in melanoma cells that are not MITF dependent [92], indicating that BRG1 and the related BRM ATPase coactivate multiple transcriptional regulatory factors in melanoma. Of particular interest, BRG1 stimulates the expression of genes that encode proteins involved in melanoma invasiveness [87]. A recent study showed that BRG1 is recruited by MITF and SOX10 to a set of MITF-associated regulatory elements at active enhancers [93]. The distinct pattern of binding by MITF, SOX10 and two other transcription factors between two BRG1-occupied nucleosomes determines a specific chromatin organization of the regulatory elements that is essential for gene expression and biological function. BRG1 also regulates the dynamics of MITF genomic occupancy. The interplay between MITF and BRG1 thus plays an essential role in transcription regulation in melanoma.

### Neuroblastoma/medulloblastoma/glioma

A limited number of studies examining BRG1 in different kinds of brain or nervous system tumors provide evidence of elevated levels of BRG1 in these tumors. In a recent study, BRG1 was found to be overexpressed in advanced neuroblastomas and associated with poor prognosis for neuroblastoma patients [94]. Reduction in BRG1 levels in neuroblastoma cell lines led to slow proliferation in culture and in mouse xenografts. Global gene expression analysis showed that BRG1 depletion mainly affected genes associated with cell growth and proliferation, cell death and survival, including components of the PI3K/AKT pathway [94]. In another study, knockout of Brg1 in a sonic hedgehog-type medulloblastoma mouse model markedly attenuated tumor formation and progression [95]. Global gene expression studies revealed that Brg1 functioned as a coregulator for key transcription factors, including Gli1, Atoh1 and REST, to control Shh-type medulloblastoma growth. Furthermore, these authors demonstrated that Brg1 controls gene expression at least in part through epigenetic mechanisms involving the regulation of histone H3K27 methylating and demethylating enzymes [95]. Note, however, that there are multiple types of human medulloblastomas; two other types have been associated with heterozygous missense or in-frame Ins/Del mutations in BRG1 [47,95]. Such mutations likely result in loss of one or more BRG1 functions, though dominant gain of function changes from these mutations remain a possible explanation for the observed phenotypes. Therefore, it is possible that BRG1 functions as a tumor driver or a tumor suppressor in the context of different types of medulloblastomas. Both benign and malignant gliomas, tumors of the glial cells, have increased levels of BRG1 relative to nontumorigenic adjacent tissue [96]. Knockdown of BRG1 in glioma cells led to reduced cell proliferation via a reduction in cyclin D1 and a reduction in *in vitro* migration and invasion, which was linked to downregulation of the matrix metalloprotease MMP2 [96].

### Colon/colorectal cancer

Colorectal cancer (CRC) results from an accumulation of genetic and epigenetic changes in colon epithelial cells, which transforms them into adenocarcinomas [97]. In primary colorectal tumors, BRG1 expression was frequently elevated, and knockdown of BRG1 reduced cell proliferation in primary tumor-derived cancer cells in culture [98]. Further analysis showed that BRG1 inhibited expression of the PTEN tumor suppressor. PTEN normally suppresses PI3K-Akt signaling in all cells; BRG1 knockdown, therefore, elevated PTEN, further reduced active components of the PI3K-AKT signaling pathway, and reduced cyclin D1 levels, all of which contributed to the slow proliferation phenotype that was observed. BRG1 overexpression, reduced or absent PTEN expression and elevated cyclin D1 levels were correlated in 35% of CRC primary tumors, suggesting misregulation of this pathway in a sizable number of CRC patients [98].

In a clinical analysis of colon cancer, BRG1 expression levels were positively correlated with cancer progression and negatively correlated with patient survival. Reduction of BRG1 levels resulted in reduced cell proliferation in culture and reduced tumor growth in orthotopic transplants [99]. Prior studies had implicated WNT3A as a regulator of colon cancer cells [100]. Further examination of primary tumors revealed a positive correlation between BRG1 and WNT3A expression. As would be predicted, BRG1 knockdown reduced WNT3A expression in cultured cells, and reintroduction of WNT3A rescued the cell proliferation defect caused by BRG1 knockdown [99].

Studies of BRG1 in CRC metastasis illustrate the complex functions of BRG1. In contrast to the work just described, loss of BRG1 promotes CRC metastasis in culture and in animal models. BRG1 expression inversely correlated with metastasis, and knockdown enhanced metastatic cancer cell mobility, migration and invasiveness through an axis of regulators involving miRNA regulation of a regulator of Wnt/β-catenin signaling [101]. Follow-up studies concluded that BRG1 reduction or loss promoted lymphangiogenesis in colorectal tumors through regulation of STAT3 activation and VEGF expression [101]. Collectively, these studies provide an intriguing contrast in the consequences of misregulation of BRG1. Additional studies will be needed to better define the oncogenic versus tumor suppressive properties of BRG1 in CRC.

### Pancreatic cancer

Pancreatic ductal adenocarcinoma (PDA), the most common form of pancreatic cancer, can arise from precursor lesions via distinct mechanisms. Included among these, precancerous lesions are pancreatic intraepithelial neoplasia, which derives from exocrine acinar cells [102], and intraductal papillary mucinous neoplasia (IPMN), which derives from pancreatic ductal epithelial cells [103]. Numerous reports indicate a tumor suppressive role for BRG1 in PDA. Examining BRG1 levels in IPMN lesions revealed that lower Brg1 expression was more frequently observed in high-grade IPMNs compared with intermediate-grade and low-grade IPMNs [104]. Additional studies identified PDA tumors containing mutations in BRG1 and other SWI/SNF subunits, albeit at low frequency [105–107]. These results seem to indicate that BRG1 has a tumor suppressor role in pancreatic cancer.

However, other work indicates that the role of BRG1 in pancreatic cancer is not necessarily clear. A study of primary tumor samples showed that expression of both BRG1 and the related BRM ATPase were correlated with disease state, and that high levels of BRM was prognostic for poor survival [108]. Another study also found high levels of BRG1 expression in primary tumor samples and that BRG1 knockdown in pancreatic cancer cell lines reduced cell proliferation in culture and in xenografts and decreased AKT signaling [72]. BRG1 knockdown also reduced chemoresistance to a specific chemotherapeutic drug. Though the mechanism was not defined, the result is consistent with our previously described studies showing that BRG1 is required for drug-induced ABC transporter induction [67].

Most pancreatic adenocarcinomas contain mutations in the *KRAS* gene that encodes a GTPase that is integral to many cellular signaling pathways [109]. Mouse modeling determined that depletion of Brg1 cooperated with mutant Kras to rapidly form IPMN-like lesions that then progressed to PDA. In contrast, loss of Brg1 inhibited mutant Kras-driven formation of PanID lesions from adult acinar cells [103]. These data indicate that BRG1 has opposing roles in the development of different precancerous lesions that lead to PDA. Subsequent studies showed even greater complexity to Brg1 function, as the same research team examined IPMN progression to PDA in detail. They determined that the ductal cells from which IPMN is derived undergo dedifferentiation as part of the tumorigenic process; Brg1 functions as a tumor suppressor at this stage to prevent dedifferentiation and tumor initiation. However, once PDA forms, Brg1 drives PDA progression by inducing an epithelial to mesenchymal transition. Therefore, Brg1 prevents and promotes pancreatic cancer in a stage-specific manner [110]. Reconciling these conflicting roles for Brg1 and exploiting this information therapeutically for PDA awaits further work.

### Other cancers

Additional tumor types where elevated BRG1 expression has been reported include gastric, prostate and intestinal cancers. Approximately 60% of the gastric carcinomas assayed showed elevated levels of BRG1 relative to non-neoplastic mucosa, and tumors at more advanced stages showed further increased levels of BRG1. Assessment of genetic alterations in BRG1 present in the tumors or in eight gastric carcinoma cell lines failed to identify any mutations, suggesting that elevated expression was associated with the development and progression of the tumor [111]. However, a different report indicated that BRG1 or one of the other SWI/SNF enzyme subunits was mutated in approximately 30% of gastric cancers [112], similar to what was reported by another group studying pancreatic cancer [106]. The reason for the discrepancy remains to be addressed. Elevated levels of BRG1 have been reported in prostate cancer, with BRG1 expression level correlating with advancement of the tumor [113]. In one of the few reported instances of successful overexpression of BRG1, these authors showed that overexpression of wildtype but not a catalytically inactive BRG1 increased prostate cancer cell line invasiveness *in vitro* [113]. Finally, mouse modeling of intestinal cancer demonstrated that knockout of Brg1 in small intestine epithelium or in the intestinal stem cell population attenuated expression of Wnt target genes and suppressed Wnt-driven tumor initiation [114], providing evidence for a tumor promoting function for Brg1.

## The impact of BRG1 in cancer

**Figure 2.**
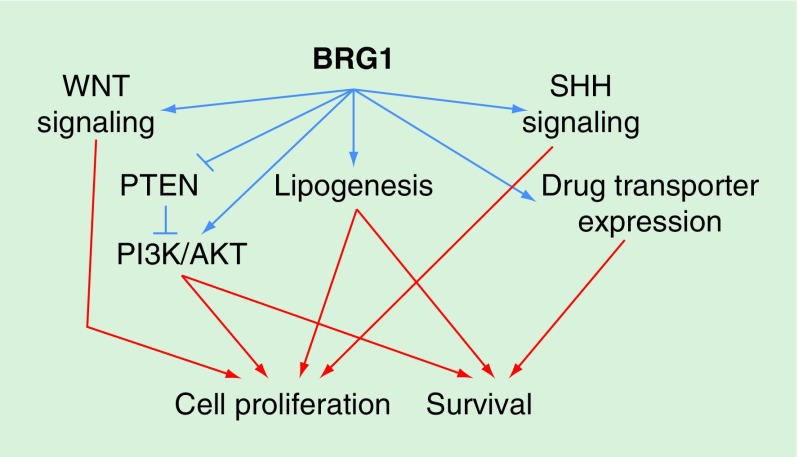
**Pathways implicated in BRG1 function in tumors and cancer cell lines showing elevated levels of BRG1.** See text for details.

Collectively, these findings reveal the influences of BRG1 on a number of signaling pathways and processes that ultimately impact cell proliferation and survival (Figure 2). The summary diagram is no doubt oversimplified; there are likely many additional pathways that are impacted by BRG1 and that converge downstream on cell proliferation and survival. Nevertheless, the findings to date have provided evidence that multiple tumor types show elevated levels of BRG1 and that targeting BRG1 suppresses cell proliferation. Thus, for certain types of cancer, BRG1 function represents a target to exploit for therapeutic purposes.

## Strategically targeting BRG1 for cancer therapy

In theory, elevated expression of any protein without mutation in a tumor cell makes that protein a potential target for therapeutic purposes. However, proteins that are ubiquitously expressed, such as BRG1, may seem to be of questionable value as targets. Nevertheless, emerging data support the idea that targeting BRG1 is a viable option. Rationales fall into two categories: targeting BRG1 as a regulator of cancer-driving pathways and inhibiting BRG1 as a method to increase the chemosensitivity of drugs already in use in the clinic.

We have discussed roles of BRG1 in specific pathways critically involved in cellular transformation. BRG1 plays a critical role in driving cancer-specific metabolic pathways in breast cancer and is linked to different signaling pathways that promote colorectal and pancreatic cancer, neuroblastoma, medulloblastoma and melanoma. Reducing BRG1 expression in tumor cells, and in some cases, orthotopic xenografts, consistently showed a reduction in cancer cell proliferation. Thus, a mechanism to deliver knockdown vectors or identification of a small molecule inhibitor of BRG1 should permit targeting of these BRG1 functions with a resulting reduction of cancer cell proliferation.

Recent reports show that reduction of BRG1 expression results in increased chemosensitivity to individual drugs that target rapidly dividing cancer cells [67,69–72,115–117]. One study demonstrated that half a dozen chemotherapeutic drugs currently used in the clinic were more effective in killing breast cancer cells in which BRG1 expression was reduced, demonstrating the generality of this targeting strategy [67]. Importantly, the mechanism for increased tumor cell killing was a dependence on BRG1 for the drug-induced activation of ABC transporter gene expression [67]. Since ABC transporters often control the import and export of chemotherapy drugs, targeting BRG1 should prevent or reduce ABC transporter induction and prevent transporter-mediated export of the drugs and treatment failure. Targeting BRG1, then, should be an effective adjuvant therapy for existing cancer treatments. Other studies linked the chemosensitivity observed upon BRG1 knockdown to deficiencies in DNA repair following exposure to DNA damaging chemotherapeutic drugs [115–117], suggesting another drug resistance mechanism that would be targeted by methods reducing BRG1 expression or function.

While methods for targeting cancer-driving proteins by siRNA and for genome editing to alleviate the effects of mutation or misexpression progress, the classic approach of identifying inhibitors of target molecules continues. Small molecule targeting of BRG1 and related proteins has already begun.

PFI-3 is a small molecule inhibitor developed by Pfizer and the Structural Genomic Consortium that specifically targets the bromodomain of family VIII bromodomain proteins, which include BRG1, BRM and the Polybromo 1 (also called BAF180) subunit of SWI/SNF enzymes. Embryonic stem cells treated with PFI-3 showed reduced stemness potential and deregulated lineage specification, with markedly enhanced trophoblast differentiation [118]. Subsequent work determined that PFI-3 impaired both myoblast and preadipocyte differentiation [119]. However, PFI-3 had no effect on cell proliferation in various human cancer cell lines, perhaps because it failed to dislodge BRG1/BRM from chromatin [67,120]. It can, therefore, be concluded that the bromodomains of BRG1, BRM and Polybromo 1 contribute to the balance between stemness and differentiation, but are not required for BRG1- or SWI/SNF enzyme-dependent cancer cell proliferation.

Active DNA-dependent ATPase A Domain inhibitor (ADAADi) is a minor byproduct of the reaction catalyzed by the bacterial aminoglycoside-3′-phosphotransferase APH (3′)-III enzyme that can be separated by chromatographic steps [121,122]. ADAADi is an inhibitor of the ATPase activity of the SWI2/SNF2 family of ATPases, and it specifically blocks nucleosome remodeling by BRG1-based SWI/SNF enzymes [121]. The activities of other related DNA-dependent ATPases, RNA-dependent ATPases and DNA-independent ATPases are not affected by ADAADi [121]. In breast cancer cells, ADAADi preferentially targets BRG1 over BRM, suggesting that some specificity may exist *in vivo*. Remarkably, treatment of breast cancer cells with ADAADi reduced cell proliferation. Furthermore, ADAADi decreased *de novo* lipid synthesis in breast cancer but not breast epithelial cells and enhanced chemotherapy drug efficacy to the same extent as BRG1 knockdown [63,67]. Thus, each of the documented BRG1-dependent attributes of breast cancer cells could be abrogated by the inhibitor. This work provides the first proof of principle that a BRG1 inhibitor can directly inhibit cancer cell proliferation as well as complement the chemotherapeutic activities of clinically relevant drugs currently used for patient treatment. Efforts to identify the active molecule in ADAADi and the discovery of additional BRG1 inhibitors will provide the oncology community with novel tools to augment current therapeutic approaches.

## Future perspective

The BRG1 ATPase that drives some of the SWI/SNF chromatin remodeling enzymes is an intriguing contributor to cancer. Its basic biochemical function, to disrupt histone:DNA contacts to alter nucleosome structure and/or positioning, makes its biological relevance subject to its immediate cellular context. The chromatin remodeling activity can be used to promote the function of transcriptional activators as well as repressors and to facilitate replication, recombination and repair. Perhaps it is not so surprising, then, that in the context of cancer initiation and progression, BRG1 activity can cooperate with factors promoting tumor suppression as well as oncogenic factors promoting unregulated cell proliferation.

Surprising or not, this reality introduces significant complexity into understanding BRG1 function and mechanisms of action. Work on BRG1 and other SWI/SNF enzyme subunits as tumor suppressors based on their mutation or loss of expression in some cancers continues, but there is now ample evidence that elevated BRG1 expression is associated with other cancer types and, at least in some cases, with poor prognosis. Consequently, there are studies showing that reintroduction of BRG1 into tumor cells lacking BRG1 inhibits tumor cell proliferation as well as studies showing that proliferation of other tumor cell types can be inhibited by BRG1 knockdown or depletion. The recent studies of pancreatic cancer that showed both tumor suppressive and tumor-promoting activities at different tumor stages demonstrates that BRG1 can have opposing functions even within the same cancer [103,110]. This reinforces the concept that BRG1 function is highly context dependent.

Many of the studies showing that alteration of BRG1 expression impact tumor cell proliferation include hypotheses stating that modulating BRG1 expression may be a future therapeutic approach, but the conundrum is clear. Any therapeutic strategy is going to be particularly dependent on precise delivery of the BRG1 ‘modulator’ to the tumor. A further complication is the possible, or perhaps likely, effect of any therapeutic strategy targeting BRG1 on the other SWI/SNF enzyme subunits. Limited data exist on the relationship between BRG1 and BRM expression, though it has been documented that knockdown of one ATPase can lead to increased expression of the other [25,59,61]. The concept of functional compensation of one ATPase for the other provides additional hurdles in developing treatments based on either enzyme. In addition, there is still much to be learned about how manipulating one subunit will impact the expression of the other subunits, assembly of the enzyme complex and functional consequences on individual subunits and the enzyme itself.

Nevertheless, the community finds itself at the onset of an exciting new phase of research. The idea that a biologic or chemical inhibitor of BRG1 could be used to improve cancer therapy is tantalizing. To date, it is apparent that one bromodomain inhibitor, though clearly modulating SWI/SNF function in developmental processes, had no impact on the proliferation of any of a number of different tumor cell types [67,118–120]. Whether other bromodomain inhibitors are similarly ineffective remains to be determined, it has been reported that ectopic expression of the BRG1 bromodomain increased the radiosensitivity of cancer cells [123]. An inhibitor of the BRG1 ATPase domain, however, showed dramatic effects in inhibiting cancer cell proliferation and in inhibiting BRG1-specific underlying mechanisms driving increased lipid synthesis [63], giving credence to the idea that the ATPase domain, despite its conservation, is a viable target. The additional finding that the ATPase inhibitor increased chemosensitivity of known chemotherapeutic drugs [67], combined with similar results based on BRG1 knockdown [67,69–72], raises the possibility that targeting BRG1 could complement existing cytotoxic drug use. The possibility of targeting other functional domains in BRG1 (Figure 1) is unknown. Undoubtedly the community will continue searching for additional molecules that can inhibit cancer cell proliferation, which we expect will be successful. The real challenge, and of course, interest, will be in the subsequent efforts to determine whether these molecules can be utilized beyond cultured cancer cell models to effectively and specifically target BRG1 in patient tumors.

**Executive summary**

- BRG1, a catalytic subunit of the SWI/SNF family of ATP-dependent chromatin remodeling enzymes, has tumor suppressor activities, but is highly expressed in some tumor types without mutation.
- Characterization of BRG1 function in tumors with elevated BRG1 levels shows effects on signaling pathways that result in increased cancer cell proliferation and survival.
- Knockdown of BRG1 in tumor cell types that show elevated BRG1 levels suppresses proliferation and other cancer cell properties.
- An inhibitor of the ATPase activity of BRG1 similarly suppresses cancer cell proliferation and other cancer cell phenotypes and provides proof of principle that targeting BRG1 could be of therapeutic value.
- BRG1 knockdown or the BRG1 inhibitor increases chemosensitivity to cytotoxic drugs used clinically for cancer treatment, suggesting that BRG1 targeting could be a successful adjuvant therapy to existing chemotherapeutic approaches.
